# Gamma-glutamyltransferase and risk of cardiovascular mortality: A dose-response meta-analysis of prospective cohort studies

**DOI:** 10.1371/journal.pone.0172631

**Published:** 2017-02-23

**Authors:** Junna Wang, Dandan Zhang, Rongzhong Huang, Xingsheng Li, Wenxiang Huang

**Affiliations:** 1 Department of Infectious Diseases, The First Affiliated Hospital of Chongqing Medical University, Chongqing, China; 2 Department of Gerontology, The Second Affiliated Hospital of Chongqing Medical University, Chongqing, China; 3 Department of Rehabilitation Medicine, The Second Affiliated Hospital of Chongqing Medical University, Chongqing, China; Kurume University School of Medicine, JAPAN

## Abstract

**Background:**

Serum gamma-glutamyltransferase (GGT) elevation likely contributes to cardiovascular (CV) mortality, however it has remained unknown whether a dose-response relationship exists between serum GGT and CV mortality.

**Methods:**

We searched the PubMed, EMBASE, and Cochrane library databases for prospective cohort studies published up to October 2, 2016. Summary hazard ratios (HRs) with their corresponding 95% confidence intervals (CIs) were calculated using a fixed effects model.

**Findings:**

Nine prospective studies, including 527,589 participants and more than 7,011 cases, were included in this meta-analysis. For the moderate, high, and highest levels of GGT, the pooled HRs of CV mortality were 1.11 (95% CI = 1.04–1.19), 1.29 (95% CI = 1.21–1.38) and 1.59 (95% CI = 1.47–1.72), respectively (all p < 0.05 as compared to the lowest levels of GGT). Additionally, the HR per incremental increase of GGT by 10 U/L was 1.10 (95% CI = 1.08–1.11). Evidence of a positive relationship with nonlinear trend for GGT elevation with CV mortality in females was found (P = 0.04 for nonlinearity). However, a linear model was better fit to illustrate the GGT-CV mortality among males (P = 0.304 for nonlinearity).

**Conclusions:**

These findings indicate that serum GGT activity within the reference interval is positively associated with increased risk of CV mortality in a dose-response manner.

## Introduction

Cardiovascular disease (CVD) is one of the leading causes of mortality in developed countries and responsible for an estimated 17.5 million annual deaths in the world [1], representing as much as 60% of all deaths in regions such as Eastern Europe [2]. Despite age-standardized death rates from CVDs are estimated to be steadily decreasing for decades in the world as a whole, nonetheless, population growth and aging may lead to increase the absolute burden of CVDs [3]. Therefore, early prognosis and effective primary prevention are essential to lower the burden of this fatal disease, and identification of modifiable and biological factors would be imperative in changing CV mortality throughout whole communities and populations.

Serum gamma-glutamyltransferase (GGT), a commonly used marker of hepatobiliary disease and excess alcohol consumption [4,5], is a plasma membrane enzyme that can degrade the circulating antioxidant glutathione (GSH) and increase intracellular GSH synthesis by assimilating and reutilizing the precursor amino acid [6,7]. Beyond its physiological functions, a large number of epidemiological studies have emerged that link serum GGT within its reference interval to the incidence of chronic conditions and diseases, including metabolic syndrome, diabetes, hypertension, body mass index, hyperlipidemia and others [8–13]. It is not understood why serum GGT levels within the normal interval would be associated with various clinical diseases. In fact, serum GTT has been shown to have pleiotropic effects ranging from antioxidant to pro-oxidant [14]. However, the precise mechanism by which serum GGT activity is predominantly antioxidant or pro-oxidant in the context of various clinical diseases is not currently understood.

Despite the mechanism being unknown, there are considerable prospective studies published reporting on the independent role of serum GGT in the pathogenesis and clinical evolution of cardiovascular diseases [15,16] and CV mortality in the general population [17–24]. However, individual studies regarding the association between serum GGT levels and increased risk of CV mortality have been inconsistent. Findings from studies of the third US National Health and Nutrition Examination Survey showed that the risk of cardiovascular mortality for persons with elevated GGT was diminished and no longer statistically significant after controlling numerous CVD risk factors among 14,950 adult participants [25]. Similarly, no significant relationships between serum GGT and CV mortality were observed in 2,724 Japanese men, for whom the prevalence of smoking and drinking is high [26]. Therefore, the predictive role of serum GGT in monitoring CV mortality is still controversial, and the evolving debate is focused on whether established cardiovascular risk factors may attribute to the increased risk of CV mortality. Previous meta-analysis described that baseline levels of GGT were associated with an increased future risk of CV and all-cause mortality [27,28], however, few of these studies established a dose-response of GGT exposure associated with the risk increase or determined the shape of dose-response curve to find whether it is a linear relation, saturation or U-shaped curve relation between GGT exposure level and CV mortality risk. Additionally, numerous new studies have been reported in recent years [21–23], therefore, it is meaningful to clarify these contradictory results between serum GGT and the risk of CV mortality and more precisely evaluate the shape of dose-response association between serum GGT and CV mortality.

## Materials and methods

### Search strategy

We followed the Preferred Reporting Items for Systematic Reviews and Meta-analyses (PRISMA) guideline [29] for performing and reporting corresponding results in this meta-analysis (S1 Appendix). We systematically searched all published articles indexed in PubMed, EMBASE and Cochrane Library before October 2, 2016 without language or time limitations. The following medical subject headings were used for searching the relevant literatures: (Gamma- glutamyltransferase OR GGT OR liver enzymes OR γ-Glutamyltransferase OR gamma-GT OR γ-GT OR nonalcoholic fatty liver disease) and (cohort OR observational OR prospective OR follow-up OR longitudinal) and (cardiovascular mortality OR cardiovascular disease OR myocardial infarction OR ischemic heart disease OR coronary artery disease OR heart diseases OR coronary disease OR mortality OR CVD OR death OR heart death OR sudden death OR cause of death OR all-cause mortality OR cardiac death OR CV death OR deaths) (S2 Appendix). Additionally, we contacted the original authors to obtain extra information if necessary, and reviewed reference lists of other relevant studies and pertinent reviews to identify works that were not found in the database search.

### Eligibility criteria

The included studies in the meta-analysis had to meet the following criteria: 1) have a prospective cohort design (eg, not cross-sectional design, case-control design, retrospective cohort design, literature reviews and experimental design); 2) subjects enrolled in the study at baseline were free of any pre-existing diseases, especially cardiovascular diseases in the general population; 3) the exposure of interest was serum GGT concentration and the corresponding categories were more than 2 levels; 4) the outcome of interest was CV mortality; 5) Reported adjusted HRs or relative risks (RRs) with 95% CIs at least three quantitative GGT categories, or provided the number of cases and total participants or person-years for each category of serum GGT levels; 6) the reported HRs or RRs had been adjusted at least for age and gender; 7) the duration of follow-up was more than five years.

### Data extraction and validity assessment

One investigator (J.N.W) extracted the data from the eligible studies, another investigator (D.D.Z) validated the data for accuracy independently with a standardized form as follows: first author’s name, publication year, country of the participants, name of study or source of participants, baseline survey period, follow-up time (year), age at recruitment (mean or range), gender (female, male or both combined), mean or median range of GGT levels in each category, the size of observational population and CV mortality cases for each GGT levels, values of HRs or RRs with their 95% CIs by quantile, and adjusted potential confounders. Additionally, if the articles presented the data separately by gender, we treated them as two independent studies and extracted the data separately. When diverse adjustments were provided, we extracted the HRs (95% CIs) with the most confounders adjusted. For studies with published data in people with and without diabetes separately, we just extracted the results in subjects without diabetes. The summary data of each identified work in our meta-analysis were presented in Table 1. The quality of included studies was assessed by the Newcastle-Ottawa Quality Assessment Scale for cohort studies [30].

**Table 1:** The basic characteristics of the nine cohort prospective studies included in meta-analysis.

Study	Region	Name of study or source of participants	Year of Baseline survey	Gender (female/ Male/Both)	Follow-up (mean± SD) (yr)	Age or range mean (yr)	HR/RR	Case/Total	GGT Level (U/L)	95%CI	Adjusted confounders	Study quality
Kengne A P, 2012 [20]	Britain	HSE/SHeS	1994–2009	M	10.1	53.8	HR	402/7866	12 18 25 49	1 0.95 (0.78–1.16) 0.94 (0.76–1.16) 1.46 (1.22–1.75)	Age and sex	8
				F				317/9403	11 17 25 49	1 1.04 (0.82–1.32) 1.29 (1.03–1.61) 1.77 (1.47–2.13)		
Ruttmann E, 2005 [17]	Austria	VHM&PP	1985–2001	M	10.1±5.0	41.8	HR	1571/74830	<14 14–27 28–41 42–55 ≥56	1 1.17 (1.02–1.33) 1.28 (1.08–1.53) 1.39 (1.09–1.78) 1.64 (1.35–2.0)	Age, body mass index, systolic blood pressure, cholesterol, triglycerides, glucose, smoking, work status, and year of examination.	9
				F	10.8±4.9	42	HR	1455/89114	<9 9–17 18–26 27–35 ≥36	1 1.04 (0.88–1.22) 1.35 (1.11–1.64) 1.46 (1.14–1.88) 1.51 (1.21–1.89)		
Wannamethee S G,2008 [18]	Britain	BRHS	1978–2004	M	24	40–59	RR	1043/6997	<11 11–14 15–21 ≥22	1 1.10 (0.91,1.32) 1.13 (0.93,1.37) 1.40 (1.16,1.70)	Age, social class, smoking, alcohol intake, physical activity, pre-existing evidence of undiagnosed CHD, BMI, systolic blood pressure, cholesterol, blood glucose and HDL-cholesterol.	9
Breitling L P, 2011[19]	Germany	WCB	1986–1992	M	17	25–64	HR	507/19090	<11 11–14 15–21 22–38 ≥39	1 1.07 (0.71–1.62) 1.27 (0.87–1.86) 1.61 (1.11–2.35) 2.02 (1.39–2.94)	Age, Nationality, DM, IHD, occupation, HT, BMI, smoking, elevated blood glucose, triglycerides, cholesterol, alcohol.	8
Koehler E M, 2014 [21]	Netherlands	Rotterdam	1990–2009	Both	19.5	70.3	HR	672/5186	GGT(1) GGT(2) GGT(3) GGT(4) GGT(5)	1 1.40 (1.10,1.77) 1.58 (1.24,2.01) 1.47 (1.14,1.91) 2.07 (1.43,2.99)	Age, sex, education, smoking status, alcohol intake, BMI, diabetes mellitus, hypertension, and total cholesterol levels.	8
Sung K C, 2015 [22]	Korea	HSP	2002–2009	Both	7	40.2	HR	178/260260	1–12 13–19 20–34 ≥35	1 0.92 (0.50–1.70) 1.21 (0.67–2.20) 1.35 (0.72–2.56)	Age, sex, smoking status, alcohol intake, diabetes, LDL cholesterol, history of heart disease, hypertension, history of stroke and fatty liver, HDL cholesterol, regular exercise, and BMI.	8
Li Y, 2016 [23]	Japan	EPOCH-JAPAN	NP	M	8.7	40–79	HR	361/15987	1–16 17–24 25–40 41–837	1 1.32(0.99–1.76) 1.33(0.96–1.82) 1.39(0.97–1.99)	Age, drinking status, smoking status, BMI, systolic blood pressure, serum triglycerides levels, serum total cholesterol levels, aspartate aminotransferase, alanine aminotransferase.	9
				F				340/25053	1–9 10–13 14–18 19–435	1 1.04(0.73–1.47) 1.01(0.69–1.48) 1.58(1.08–2.29)		
Haring R, 2009 [24]	Germany	SHIP	NP	M	7.3	20–79	HR	NP	Q1 Q2 Q3 Q4 Q5	1 1.67 (0.72–3.88) 1.86 (0.81–4.30) 1.48 (0.61–3.60) 2.80 (1.24–6.31)	Age in decades, waist circumference, alcohol consumption, physical activity, educational level, civil status, equalized income, and Functional Comorbidity Index.	9
				F				NP	Q1 Q2 Q3 Q4 Q5	1 1.52 (0.36–6.43) 1.22 (0.28–5.33) 1.85 (0.47–7.21) 2.34 (0.61–8.96)		
Hozawa A, 2007 [26]	Japan	NIPPON DATA	1990–2000	M	9.6	52.5	HR	83/2724	1–12 13–24 25–49 50–468	1 0.99 (0.48–2.04) 0.77 (0.34–1.76) 0.84 (0.30–2.39)	Age, alcohol consumption, cigarette, smoking, GOT, GPT, body mass index, HDL-cholesterol, total-cholesterol, triglyceride, habitual exercise, systolic BP, use of antihypertensive medication and diabetes.	9
				F			HR	82/4122	1–12 13–24 25–49 50–295	1 1.16 (0.68–1.98) 1.89 (0.95–3.75) 2.97 (1.06–8.34)		

Abbreviations: M, male; F, female; SD, standard deviation; BMI, Body mass index; NP, not provided; Q, Quintile; BP, blood pressure; GOT, Glutamic-oxaloacetic transaminase; GPT, Glutamicpyruvic transaminase; HDL, High-density lipoprotein; LDL, Low-density lipoprotein; CHD, coronary heart disease; IHD, ischemic heart disease; HT, hypertension; DM, Diabetes mellitus; VHM&PP, Vorarlberg Health Monitoring and Promotion Program; NIPPON DATA, National Integrated Project for Prospective Observation of Non-communicable Disease and Its Trends in the Aged; SHIP, Study of Health in Pomerania; EPOCH-JAPAN, The Evidence for Cardiovascular Prevention from Observational Cohorts; BRHS, British Regional Heart Study; HSE, Health Survey of England; SHeS, Scottish Health Survey; WCB, Workmen’s Compensation Board; HSP, Health screening program; HRs, hazard ratios; RRs, relative risks; CIs, confidence intervals.

### Data synthesis and statistical analysis

We conducted separate meta-analyses for different levels of GGT concentration categorized into four levels as in a meta-analysis of Coffee Consumption and Risk of Cardiovascular Disease [31]. We calculated the pooled risks and 95% CIs of CV mortality by the general variance-based method that requires information on the RRs or HRs and the 95% CIs for each study [32]. We separated the GGT exposure levels into four groups, including the lowest, the moderate, the high, and the highest groups. In each included study, we considered the lowest and the highest GGT categories to be the lowest and highest groups, respectively. Therefore, if the number of GGT exposure categories was four, the second and third categories corresponded to the moderate and the high groups. However, if the original studies had five exposure categories, we pooled the HRs of the third and the fourth categories with inverse variance weight and used combined estimate for the high group.

To identify whether there is a potential nonlinear relationship between GGT levels and CV mortality, a two-stage dose-response meta-analysis was performed. We used the generalized least-squares (GLST) calculation described by Orsini and colleagues [33] and multivariate maximum likelihood methods to estimate the nonlinear trends in the effect, applying 4 fixed knots at the 5%, 35%, 65%, and 95% percentiles of GGT exposure level [34–36]. At the first stage, we constructed study-specific slopes (linear trends) and 95% CIs for every 10% increase in GGT exposure level within each study using the fixed effects model. At the second stage, we combined the study-specific estimates to obtain the summary risk estimates assuming a random-effects model. Calculations for the dose-response meta-analysis included the distributions of cases and person years, median or mean of serum GGT levels for each comparison group, and adjusted HRs with corresponding 95% CIs for more than two categories of GGT exposure. The median level of serum GGT for each category was assigned to each corresponding HR estimate. We designated the midpoint of the lower and upper boundaries as the value of GGT exposure. When the upper boundary for the highest categories of GGT exposure was not provided, we calculate the value of exposure by the lower boundary multiplying 1.5 [37]. If the lowest category was open-ended, we assumed it to be zero [38]. In order to examine whether a nonlinear models exists between GGT and CV mortality, a P value was obtained from the test of the null hypothesis that the coefficient of the second spline is equal to zero. For the 3 articles [17,18,20] that did not provide person years for each GGT category, we calculated data for inclusion in the analysis from mean follow-up duration and number of subjects. Two studies [21,24] were excluded in the dose-response meta analyses because they did not report the number of subjects (total number of CV mortality or corresponding GGT exposure level).

Furthermore, we performed subgroup analysis according to study location, gender, age, follow-up time, sample size, adjustment of diabetes mellitus, body mass index (BMI) and alcohol intake. To check whether the results could have been affected markedly by a single study, diverse sensitivity analyses were carried out by removing one study at a time and recalculating the pooled estimates for the remainder of the studies.

Heterogeneity among studies was quantitatively evaluated using the chi-square-based Q test (P < 0.1, significant heterogeneity) and I^2^ statistic (I^2^ < 30%, low heterogeneity; I^2^ = 30–50%, moderate heterogeneity; I^2^ > 50%, substantial heterogeneity) [39]. A random-effect model was applied if the heterogeneity was significant, otherwise, a fixed-effect model was conducted [40]. Potential publication bias was tested by the application of contour-enhanced funnel plots [41], Begg’s rank correlation test [42] and Egger’s linear regression test [43] with P values less than 0.05 indicating publication bias. Statistical calculations and figures were performed using Stata 14.0 version software (Stata Corp, College Station (TX)).

## Results

### Study characteristics

**Fig 1:**
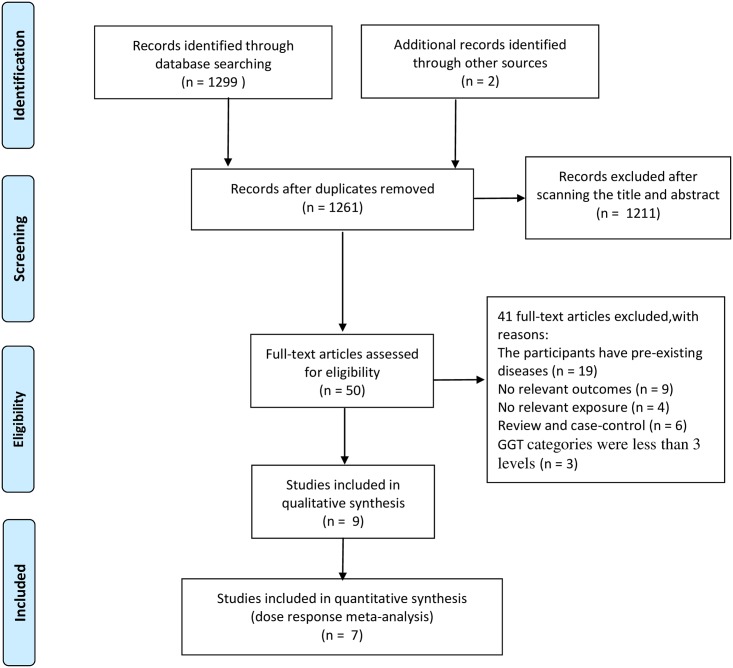
Flow diagram of eligible literature selection.

A total of nine independent prospective articles [17–24,26] containing fourteen studies reporting serum GGT and CV mortality were used in the meta-analysis, including more than 7,011 CV mortality cases identified among 527,589 participants (Fig 1). Of these, seven epidemiological articles [17–20,22,23,26] were finally included in the dose-response meta analysis, comprised of 515,446 participants (6,339 CV mortality cases). Of the nine cohort articles, three articles were conducted in Asia including two studies from Japan [23,26] and one from Korea [22], the remaining six articles were conducted in Europe, one in Austria [17], one in Netherlands [21], two in Britain [18,20] and two in Germany [19,24]. Five articles reported the effects of serum GGT on CV mortality separated by gender [17,20,23,24,26], two articles [21,22] reported their results without distinguishing by gender, and two articles [18,19] reported the results on males only. The follow-up duration was ranging from 7 to 24 years, and sample sizes ranging from >5,000 to >260,000 subjects. The age of participants at baseline varied from 20–79 years. The incidence of CV mortality in enrolled studies ranged from 2.1%-5.1% in males, which was always higher than corresponding data in females (1.4%-3.4%) from the same cohort. In all studies, CV-specific mortality was assessed by medical records or death certificates based on International Classification of Diseases (ICD) codes-9,10. All except one studies [20] provided multivariate-adjusted risk estimates (e.g., age, sex, body mass index, smoking, cholesterol, et al.). Each of the eligible articles was awarded at least eight stars according to the Newcastle-Ottawa Quality Assessment Scale for cohort studies.

### GGT and CV mortality incidence

#### High versus low

**Fig 2:**
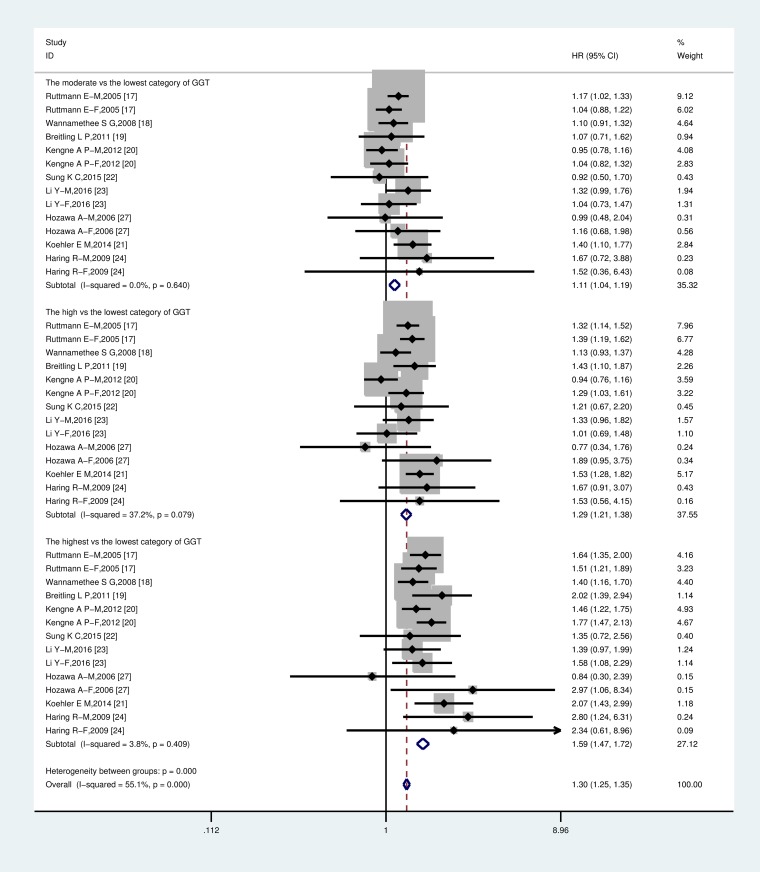
Forest plot of hazard ratios of the moderate, the high and the highest vs. the lowest category of serum GGT with CV mortality risk. The squares represent the risk estimate for each individual study, with the area reflecting the weight assigned to the study. The horizontal line across each square represents the 95% confidence interval. The diamond represents the summary risk estimate, with width representing 95% confidence interval. The pooled hazard ratios were calculated by a fixed-effect model for all p values for heterogeneity >0.05. Abbreviation: CI, confidence interval; HR, hazard ratio. The hazard ratios were adjusted for potential confounders.

Compared to the subjects with the lowest GGT categories in fourteen studies, the pooled HRs (95% CI) for CV mortality incidence were 1.11 (95% CI: 1.04–1.19, P = 0.002) for the moderate, 1.29 (95% CI: 1.21–1.38, P = 0.000) for the high and 1.59 (95% CI: 1.47–1.72, P = 0.000) for the highest categories of GGT levels. The P values for heterogeneity of the Q test in the above analyses were 0.64, 0.079 and 0.409, respectively, moderate heterogeneity was only upon analyzing the high versus the lowest categories of GGT (Fig 2).

#### Dose-response analyses

**Fig 3:**
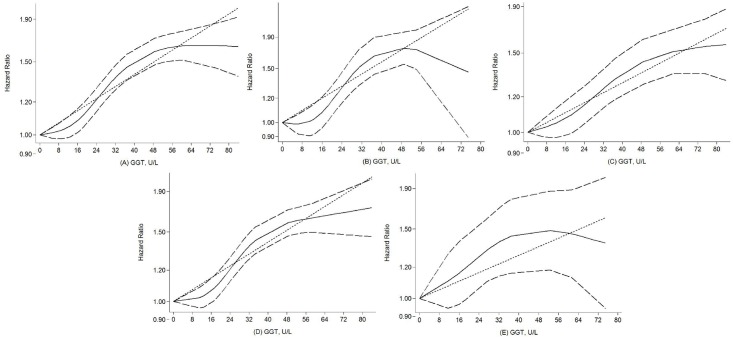
Dose-response relationship between serum GGT levels and risk of CV-mortality in prospective studies. Restricted cubic splines and generalized least squares dose-response models on evaluation of association between GGT and risk of CV mortality. (A) Overall analysis; (B) females; (C) males; (D) Europe; (E) Asia. The solid line represents the fitted hazard Ratio curve compared to the subgroup with the lowest mean levels of serum GGT, and Lines with long dashes represent 95% CI of this risk by restricted cubic splines model. Lines with short dashes represent the weighted regression index compared to subgroup with lowest mean levels of serum GGT by generalized least squares model.

**Fig 4:**
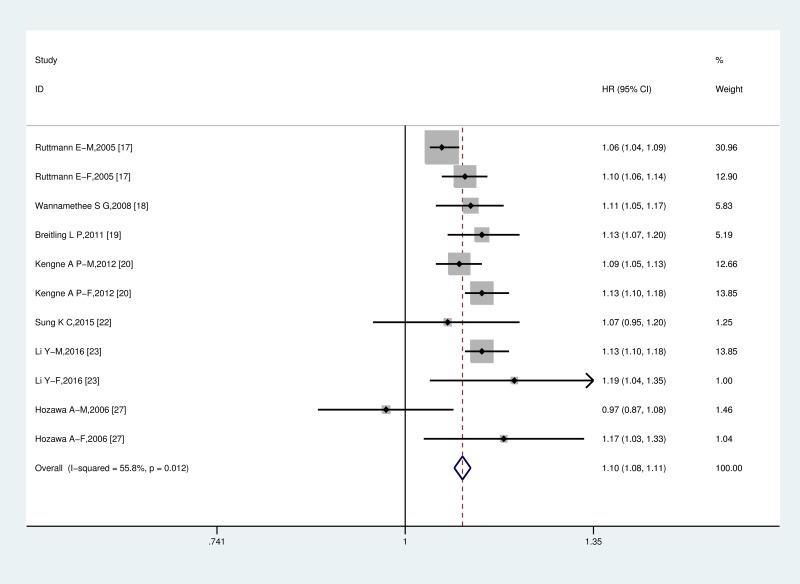
The two-stage dose-response meta-analysis on serum GGT and CV mortality. The squares represent the risk estimate for each individual study, with the area reflecting the weight assigned to the study. The horizontal line across each square represents the 95% confidence interval. The diamond represents the summary risk estimate, with width representing 95% confidence interval. CI, confidence interval; HR, hazard ratio.

We found a significant dose-response association between serum GGT levels and the incidence of CV mortality. Using a restricted cubic splines model, there was no evidence of departure from nonlinearity among the data from the included studies (P_nonlinearity_ < 0.001. see Fig 3A). The nonlinear dose-response trends showed a significantly increased risk of CV mortality with increasing serum GGT concentrations up to 52.5U/L and then the hazard ratios stabilized approximately at the value of 1.6. An approximate 10% (HR = 1.10, 95% CI = 1.08–1.11) increase in cardiovascular-related deaths occurred per 10 U/L increment in GGT elevation, with substantial heterogeneity (I^2^ = 55.8%, P = 0.012) (Fig 4). However, when we further restricted the analyses to studies among females, the shape of the curve became steeper and there was evidence of a significantly increased risk within the high normal range of GGT (P = 0.04. Fig 3B). In contrast, there was no nonlinear relationship between GGT and CV mortality risk among males (P = 0.304. Fig 3C), indicating that a linear model was better fit to illustrate the GGT-CV mortality among males. The study-specific HRs per 10 U/L increase in serum GGT concentration are presented in S1 Table. When stratified by geographical location, examination of the dose-response analysis suggested that nonlinearity relationship also existed in the European populations (P for nonlinearity = 0.003; Fig 3D). Nonetheless, in analysis restricted to Asian populations, we did not detect a significant nonlinear dose-response relationship after pooling the results (P for nonlinearity = 0.238; Fig 3E). A weighted linear relationship was demonstrated (P = 0.003).

### Subgroup and sensitivity analysis

To examine the stability of the primary results, we carried out subgroup analyses across the 11 studies [17–20,22,23,26] included in our dose-response analysis. The association did not appear to be modified by sample size, follow-up duration, geographic region of the study cohort or adjusted for diabetes mellitus (Table 2). Restricting the analysis to studies that adjusted for BMI did not alter the results. However, the results should be explained carefully as all of the original studies except one study [20] adjusted for BMI. Alcohol consumption was a suggestive effect modifier of the relationship between GGT and CV mortality, where specifically, studies that adjusted for alcohol consumption observed a somewhat stronger positive association (HR per 10 U/L = 1.12; 95% CI, 1.09–1.15), compared to studies that did not adjust for alcohol consumption (HR per 10 U/L = 1.09; 95% CI, 1.07–1.10), with P_interaction_ = 0.029. In addition, there was significant difference between subgroups when stratified by gender and age (P < 0.05 for inter-subgroup comparison). The results of sensitivity analyses are present in S2 Table. Ignoring a single study and reevaluating summarized HRs of the remaining studies in turn did not significantly alter the initial relationship of GGT and CV mortality risk. These findings confirm the reliability of our results.

**Table 2:** Subgroup analyses of pooled Hazard Ratios (HRs) of CV mortality per 10 U/L increase in GGT level.

Subgroup	Number of studies	HR(95%CI)	P value	Test for heterogenity*		
				I^2^(%)	P_heterogensity_	P_interaction_
All studies	11	1.10 (1.08, 1.11)	0	58.9	0.007	
sex						
Male	6	1.09 (1.07, 1.10)	0	70.4	0.005	0.039
Female	4	1.12 (1.09, 1.15)	0	0.10	0.391	
Follow-up duration						
≥10y	6	1.09 (1.08, 1.11)	0	60.0	0.029	0.109
<10y	5	1.12 (1.09, 1.16)	0	56.9	0.054	
Sample size						
≥10000	6	1.09 (1.07, 1.11)	0	64.1	0.016	0.249
<10000	5	1.11 (1.08, 1.13)	0	56.0	0.059	
Age						
≥50 y	5	1.11 (1.08, 1.13)	0	57.5	0.052	0.039
<50y	2	1.07 (1.05, 1.09)	0	50.0	0.157	
Study location						
Europe	6	1.09 (1.08, 1.11)	0	60.0	0.029	0.109
Asia	5	1.12 (1.09, 1.16)	0	56.9	0.054	
Adjusted for DM						
Yes	4	1.10 (1.06, 1.15)	0	60.7	0.054	0.776
No	7	1.09 (1.08, 1.11)	0	63.9	0.011	
Adjusted for alcohol						
Yes	7	1.12 (1.09, 1.15)	0	37.4	0.143	0.029
No	4	1.09 (1.07, 1.10)	0	69.9	0.019	
Adjusted for BMI						
Yes	9	1.09(1.07,1.11)	0	60.2	0.010	
No	2	1.11(1.09,1.14)	0	56.2	0.131	0.160

*For the test of heterogeneity in each subgroup, we also calculated the I^2^ statistic, and 50% was regarded as the cutoff point for non-significant and significant levels. Moderate heterogeneity was detected in our dose-response meta-analysis and heterogeneity was found when stratified by sex, age and adjustment for alcohol.

Abbreviations: HRs, hazard ratios; RRs, relative risks; CIs, confidence intervals; DM, Diabetes mellitus; BMI, Body mass index.

### Publication bias analysis

There was no evidence of publication bias for any association as revealed by Begg’s test and Egger’s test (all P > 0.05).

## Discussion

We performed a meta-analysis of the moderate, the high, and the highest levels of serum GGT compared to the lowest level of serum GGT and dose-response relationship between GGT and the incidence of CV mortality. There was evidence of a nonlinear positive dose-response relationship between GGT and CV mortality after pooling seven articles including 515,446 participants and 6,339 cases of CV mortality. Notably, a flat association was observed in the lowest range of GGT levels < 13U/L, thereafter the shape of dose-response curve was steeper with serum GGT levels 13 -< 52.5U/L. Furthermore, the increases in risk somewhat flattened at above 52.5U/L GGT. For the dose-response meta-analysis, increases of 10U/L of circulating GGT levels were associated with a 10% increase in the risk of CV mortality. Sensitivity analysis was conducted further and did not significantly alter the result. No evidence of heterogeneity or publication bias was detected across the 9 prospective articles. Compared with the lower categories, those of the moderate or highest GGT activity categories did have a significantly increased risk of CV mortality in all participants combined and in subgroups. There is an incremental increase in CV mortality of approximately 9% for males and 12% for females per 10 U/L of GGT elevation, this association seems to be stronger for females than males. However, the current findings should be interpreted critically, considering that the GGT level of comparison groups was quite different.

The results from the British Women’s Heart and Health Study indicated that GGT activity did not have a causal role in the incidence of coronary heart disease (CHD), independent of alcohol intake [44]. However, a prospective study in the Japanese population found that the serum GGT level was predictive of mortality from cardiovascular mortality among females for whom the prevalence of ever-drinkers was very low, but no associations were seen among males [26]. The reason for the gender difference remains unclear, and the authors suggested that the difficulty in controlling for the effects of alcohol consumption in males might partly account for the gender difference. One possible explanation for this result could be that moderate alcohol consumption reduces the risk of atherosclerosis by increasing plasma concentrations of high-density lipoprotein (HDL) cholesterol, a well-established major protective factor against CHD [45]. In addition, γ-GT is recently considered to be an oxidative stress marker. A Japan study reported that a positive correlation was observed between the circulating oxidative status and serum γ-GT levels at active estrogen stage among subjectively healthy women, which may indicate a different oxidative stress condition by menopausal stage [46]. Similarly, a Norway study confirmed that an increase in γ-GT was positively correlated with oral contraceptive use and menopause [47]. However, whether estrogen level plays a critical role in gender differences between GGT levels and CV mortality risk is still far from clear and requires further data to clarify the mechanism.

Several researchers have also reported that the association between serum GGT activity and CV mortality appears to be stronger in younger versus older subjects [17,18,20,22]. The Minnesota Heart Survey Epidemiologic Follow-up Study reported that the serum GGT concentration was a predictor for mortality from cardiovascular disease among populations aged less than 70 years, but no associations were seen among subjects aged more than or equal to 70 years [48]. Similar age interactions were noted by Wannamethee et al. in their analyses of the British Regional Heart Study [18]. However, recent reports supported positive associations between GGT and risk of CV mortality separately in the elderly where the mean age was 70 years [21,49]. Further studies are required to clarify the association in younger individuals.

Similar to previous meta-analyses [27,28], our dose-response meta-analysis found that the incidence risk of CV mortality increased with the elevation of serum GGT concentration, implying that serum GGT is a dose-dependent risk factor for the incidence of CV mortality. Several possible mechanisms could explain this association between GGT activity and CV mortality. There is growing evidence that GGT levels may be indirectly linked to atherosclerosis via coexistent oxidative stress [50], one of the most important contributors to vascular injury. GGT has been found within carotid and coronary plaques, colocalized with oxidized lipids and CD68+ foam cells [51–52], which may promote iron-dependent oxidation of low-density lipoprotein (LDL) and lead to further development of atherosclerotic plaques by influencing plaque evolution and rupture [4,53]. In addition, GGT is associated with subclinical microinflammatory response by modulating mediators such as leukotrienes [54] and S-nitrosoglutathione (GSNO) [55]. Findings from the cross-sectional analysis performed in the Multi-Ethnic Study of Atherosclerosis (MESA) cohort showed that evidence of significant linear trends for increasing trends in biomarkers of arteriosclerosis with graded increases in GGT, including oxidized low-density lipoproteins (oxLDL), interleukin-6 (IL-6), C-reactive protein (CRP), and soluble intercellular adhesion molecule-1 (sICAM-1)[56]. An Italy study indicated that macrophages may contribute to plaque b-GGT accumulation under inflammatory conditions, the heaviest of the four GGT fractions identified in plasma, which in turn play a role in the progression of atherosclerosis by modulating inflammatory processes in the plaque environment [57]. Epidemiologic evidence have shown that serum GGT levels are associated with coronary flow reserve (CFR) [58–60], arterial stiffness [61] and coronary artery calcification [62]. For instance, a Turkey study confirmed that serum GGT levels were inversely correlated with the coronary flow reserve (CFR) in normal individuals, which reflect coronary microvascular function and have been shown to be early manifestations of atherosclerosis and coronary artery disease [58–60]. Moreover, growing numbers of studies have revealed a strong association of GGT with various atherosclerotic risk factors, such as hypertension, metabolic syndrome, diabetes and others [8–10]. However, most of the studies were from epidemiologic data and to date the original articles are still limited.

Based on the RR or HR values for every dose category in each study included in the dose-response analysis, the association between serum GGT activity and the risk of CV mortality was significant in 1 study [17] and was mixed in the other 10 studies [17–20,22,23,26] (Table 1). In the study-specific dose-response meta-analysis using a 10 U/L incremental increase of GGT, the variation in the incidence of CV mortality was significant in 9 studies (P < 0.05) and not significant in 2 studies (P > 0.05) (S1 Table). However, our meta-analysis showed substantial significant heterogeneity across the 11 prospective studies (I^2^ = 55.8%, P = 0.012), therefore, the results should be interpreted with caution.

For subgroup analyses of the dose-response meta-analysis, the incidence of CV mortality increased with rises in GGT concentration in all subgroups with moderate to substantial heterogeneity, which did not alter much in the subgroup analyses. There are differences in serum GGT levels between males and females in our included studies. Therefore, within the subgroup analysis we examined gender as possible sources of heterogeneity. Females were more sensitive to develop CV mortality on the same GGT elevation degree than males (P < 0.05), revealing that gender might be confounding the GGT-CV mortality association. When stratified by age, our subgroup analysis revealed a stronger relationship on GGT-CV mortality in the older (P < 0.05). However, several researchers have reported that the association between serum GGT activity and CV mortality appears to be stronger in younger versus older subjects [17,18,20,22]. The reason for these seemingly inconsistent results remains unknown, though they might be driven in part by the wide range of age and the limited studies included in our subgroup stratified by age. Recently, a study in community-dwelling individuals aged 50 to 99 years suggested that excess risk of cardiovascular mortality was found in older persons with elevations in serum GGT, and the slope of the hazard curve for cardiovascular mortality is relatively flat when the age less than approximately 66 years old, and rose steeply thereafter [63], which further supported our findings.

We also examined region, number of participants, and length of follow-up as possible sources of heterogeneity, nevertheless, heterogeneity between studies did not alter much in the subgroup analyses. There is substantial evidence that alcohol consumption has been reported to be U-curve or J-curve association with CAD [64–65]. As GGT is the most common marker of excessive alcohol consumption, we conducted subgroups by adjusted alcohol consumption. Interestingly, there was some evidence of heterogeneity (P_heterogeneity_ = 0.029 for both comparisons), with a stronger association among studies that adjusted alcohol consumption, so it seems plausible that the relationship between GGT and CV mortality risk varies by alcohol consumption. In addition, GGT has been demonstrated to be positively associated with several cardiovascular disease risk factors, such as BMI [66] and diabetes mellitus [8], whereas there was no significant difference between subgroups classified by BMI and diabetes mellitus. Of course, the observed heterogeneity could be attributable to differences in environmental factors, methodological factors in design, and how the studies were conducted.

Compared with the previous meta-analyses [27], our study has several notable strengths. To explore the relationship of serum GGT concentration and CV mortality quantitatively, we performed a dose-response meta-analysis of published prospective studies and found a positive nonlinear relationship, which is a novel finding. Meanwhile, the total number of participants (n = 515,446) and CV mortality cases (n = 6,339) were sufficiently large, and the duration of follow-up was long, ranging from 7 to 24 years. As a result, the prospective cohort studies included in our meta-analysis are of high-quality, which allowed for detection of potential associations and eliminated the potential for recall and selection bias. Furthermore, we conducted subgroup analyses and sensitivity analyses to find out whether some characteristics could explain the results and evaluate robustness. No individual study was able to alter the nonlinear trend when we removed it from the dose-response meta-analysis. Therefore, the results are more reliable as they are not overly influenced by the effect of a single study.

Limitations of this meta-analysis should be mentioned. First, all included studies measured GGT level on a single occasion at baseline. Considering the within-subject variation of GGT level and measurement error, our results might be subject to regression dilution bias [67], indicating an underestimated effect size for the association of GGT levels with CV mortality. Second, several included studies used death certificate to ascertain the causes of death [17,19,20,23,24]. However, information from death certificate can be inaccurate [68]. Therefore, misclassification bias possibly affected our results on the associations between GGT levels and risks of death from cardiovascular disease. Third, the noticeable limitation of our study was the potential for bias due to the inability to fully adjust for confounders, such as CRP [69] and non-alcoholic fatty liver disease (NAFLD) [70], which might bias the results toward the exaggeration or underestimation of risk estimates, therefore, the positive effect of increasing GGT level on CV mortality could be attributed to other risk factors. Fourth, the prospective cohort studies focusing on the GGT-CV mortality association were mainly conducted in Europe, and this relationship should be evaluated in studies involving other ethnicities. Finally, our results may be influenced by language bias. Although no restrictions were imposed on language of publications to minimize this bias when searching the electronic databases, all analyzed studies were published in English.

## Conclusions

Altogether, our meta-analysis contributes to the literature by exploring the nonlinear dose-response relationship between GGT and the risk of CV mortality. These results indicated that GGT in normal range might be an individualized predictor in screening incidents of CV events. However, the potential mechanisms are still limited. Further research is necessary to further clarify the mechanisms and examine the potential modifiers of the GGT-CV mortality relationship.

## Supporting information

S1 Appendix PRISMA checklist. The PRISMA Checklist of this meta-analysis.
(DOC)

S2 Appendix Search strategy. (PDF)

S1 Table Study-specific and summary HRs for CV mortality, per 10 U/L increase in GGT level. (PDF)

S2 Table Dose-response meta-analysis on Hazard Ratios (HRs) for CV mortality per 10U/L increase in GGT, each study removed in turn. (PDF)
