# Supplementary material for: Gamma-glutamyltransferase and risk of cardiovascular mortality: A dose-response meta-analysis of prospective cohort studies
Source: PLoS One. 2017 Feb 23;12(2):e0172631. doi: 10.1371/journal.pone.0172631 (PMC5322906; doi:10.1371/journal.pone.0172631)
Supplement: S2 Appendix — (PDF) [file pone.0172631.s002.pdf]

**Pubmed**
#1 ((((((Gamma-glutamyltransferase[Title/Abstract]) OR γ-Glutamyltransferase[Title/Abstract]) OR GGT[Title/Abstract]) OR liver enzymes[Title/Abstract]) OR gamma-GT[Title/Abstract]) OR γ-GT[Title/Abstract]) OR nonalcoholic fatty liver disease[Title/Abstract]
#2 ((((cohort[Title/Abstract]) OR observational[Title/Abstract]) OR prospective[Title/Abstract]) OR follow-up[Title/Abstract]) OR longitudinal[Title/Abstract]
#3 (((((((((((((((cardiovascular mortality[Title/Abstract]) OR cardiovascular disease[Title/Abstract]) OR myocardial infarction[Title/Abstract]) OR coronary artery disease[Title/Abstract]) OR heart diseases[Title/Abstract]) OR coronary disease[Title/Abstract]) OR mortality[Title/Abstract]) OR CVD[Title/Abstract]) OR death[Title/Abstract]) OR heart death[Title/Abstract]) OR sudden death[Title/Abstract]) OR cause of death[Title/Abstract]) OR all-cause mortality[Title/Abstract]) OR cardiac death[Title/Abstract]) OR CV death[Title/Abstract]) OR deaths[Title/Abstract]) OR ischemic heart disease[Title/Abstract]
#4 #1 AND #2 AND #3

**Embase**
#1 'gamma-glutamyltransferase':ab,ti AND ([article in press]/lim OR [conference abstract]/lim OR [conference paper]/lim) AND [embase]/lim
#2 'γ-glutamyltransferase':ab,ti AND ([article in press]/lim OR [conference abstract]/lim OR [conference paper]/lim) AND [embase]/lim
#3 'ggt':ab,ti AND ([article in press]/lim OR [conference abstract]/lim OR [conference paper]/lim) AND [embase]/lim
#4 'liver enzymes':ab,ti AND ([article in press]/lim OR [conference abstract]/lim OR [conference paper]/lim) AND [embase]/lim
#5 'gamma-gt':ab,ti AND ([article in press]/lim OR [conference abstract]/lim OR [conference paper]/lim) AND [embase]/lim
#6 'nonalcoholic fatty liver disease':ab,ti AND ([article in press]/lim OR [conference abstract]/lim OR [conference paper]/lim) AND [embase]/lim
#7 'γ-gt':ab,ti AND ([article in press]/lim OR [conference abstract]/lim OR [conference paper]/lim) AND [embase]/lim
#8 #1 OR #2 OR #3 OR #4 OR #5 OR #6 OR #7
#9 'cohort':ab,ti AND ([article in press]/lim OR [conference abstract]/lim OR [conference paper]/lim) AND [embase]/lim
#10 'observational':ab,ti AND ([article in press]/lim OR [conference abstract]/lim OR [conference paper]/lim) AND [embase]/lim
#11 'prospective':ab,ti AND ([article in press]/lim OR [conference abstract]/lim OR [conference paper]/lim) AND [embase]/lim
#12 'follow-up':ab,ti AND ([article in press]/lim OR [conference abstract]/lim OR [conference paper]/lim) AND [embase]/lim
#13 'longitudinal':ab,ti AND ([article in press]/lim OR [conference abstract]/lim OR [conference paper]/lim) AND [embase]/lim
#14 #9 OR #10 OR #11 OR #12 OR #13
#15 'cardiovascular mortality':ab,ti AND ([article in press]/lim OR [conference abstract]/lim OR [conference paper]/lim) AND [embase]/lim
#16 'cardiovascular disease':ab,ti AND ([article in press]/lim OR [conference abstract]/lim OR [conference paper]/lim) AND [embase]/lim
#17 'myocardial infarction':ab,ti AND ([article in press]/lim OR [conference abstract]/lim OR [conference paper]/lim) AND [embase]/lim
#18 'coronary artery disease':ab,ti AND ([article in press]/lim OR [conference abstract]/lim OR [conference paper]/lim) AND [embase]/lim
#19 'heart diseases':ab,ti AND ([article in press]/lim OR [conference abstract]/lim OR [conference paper]/lim) AND [embase]/lim
#20 'coronary disease':ab,ti AND ([article in press]/lim OR [conference abstract]/lim OR [conference paper]/lim) AND ([chinese]/lim OR [english]/lim) AND [embase]/lim
#21 'mortality':ab,ti AND ([article in press]/lim OR [conference abstract]/lim OR [conference paper]/lim) AND [embase]/lim
#22 'cvd':ab,ti AND ([article in press]/lim OR [conference abstract]/lim OR [conference paper]/lim) AND [embase]/lim
#23 'death':ab,ti AND ([article in press]/lim OR [conference abstract]/lim OR [conference paper]/lim) AND [embase]/lim
#24 'heart death':ab,ti AND ([article in press]/lim OR [conference abstract]/lim OR [conference paper]/lim) AND [embase]/lim
#25 'sudden death':ab,ti AND ([article in press]/lim OR [conference abstract]/lim OR [conference paper]/lim) AND [embase]/lim
#26 'cause of death':ab,ti AND ([article in press]/lim OR [conference abstract]/lim OR [conference paper]/lim) AND [embase]/lim
#27 'all-cause mortality':ab,ti AND ([article in press]/lim OR [conference abstract]/lim OR [conference paper]/lim) AND [embase]/lim

#28 'cardiac death':ab,ti AND ([article in press]/lim OR [conference abstract]/lim OR [conference paper]/lim) AND [embase]/lim
#29 'cv death':ab,ti AND ([article in press]/lim OR [conference abstract]/lim OR [conference paper]/lim) AND [embase]/lim
#30 'deaths':ab,ti AND ([article in press]/lim OR [conference abstract]/lim OR [conference paper]/lim) AND [embase]/lim
#31 'ischemic heart disease':ab,ti AND ([article in press]/lim OR [conference abstract]/lim OR [conference paper]/lim) AND [embase]/lim
#32 #15 OR #16 OR #17 OR #18 OR #19 OR #20 OR #21 OR #22 OR #23 OR #24 OR #25 OR #26 OR #27 OR #28 OR #29 OR #30 OR #31
#33 #8 AND #14 AND #32

**Cochrane Library**
#1 "gamma-glutamyltransferase":ti,ab,kw or "γ-glutamyltransferase":ti,ab,kw or "GGT":ti,ab,kw or "gamma-GT":ti,ab,kw or "γ-GT":ti,ab,kw
#2 "nonalcoholic fatty liver disease":ti,ab,kw
#3 #1 OR #2
#4 "cohort study":ti,ab,kw or "observational":ti,ab,kw or "prospective":ti,ab,kw or "follow-up":ti,ab,kw or "longitudinal":ti,ab,kw
#5 cardiovascular mortality:ti,ab,kw or "cardiovascular disease":ti,ab,kw or "myocardial infarction":ti,ab,kw or "coronary artery disease":ti,ab,kw or heart diseases:ti,ab,kw
#6 "coronary disease":ti,ab,kw or "mortality":ti,ab,kw or "CVD":ti,ab,kw or "death":ti,ab,kw or heart death:ti,ab,kw
#7 "sudden death":ti,ab,kw or cause of death:ti,ab,kw or all-cause mortality:ti,ab,kw or cardiac death:ti,ab,kw or CV death:ti,ab,kw
#8 deaths:ti,ab,kw or ischemic heart disease:ti,ab,kw
#9 #5 OR #6 OR #7 OR #8
#10 #3 AND #4 AND #9
