# Supplementary material for: Gamma-glutamyltransferase and risk of cardiovascular mortality: A dose-response meta-analysis of prospective cohort studies
Source: PLoS One. 2017 Feb 23;12(2):e0172631. doi: 10.1371/journal.pone.0172631 (PMC5322906; doi:10.1371/journal.pone.0172631)
Supplement: S1 Table — (PDF) [file pone.0172631.s003.pdf]

S1 Table: Study-specific and summary HRs for CV mortality, per 10 U/L increase in GGT level.

First author, year	Gender (men %)	HR (95% CI)	P
Ruttmann E,2005 [17]	Male	1.06(1.04-1.09)	0.000
Ruttmann E,2005 [17]	Female	1.10(1.06-1.14)	0.000
Wannamethee S G,2008 [18]	Male	1.11(1.05-1.17)	0.000
Breitling L P,2011 [19]	Male	1.13(1.07-1.12)	0.000
Kengne A P,2012 [20]	Male	1.09(1.05-1.13)	0.000
Kengne A P,2012 [20]	Female	1.13(1.10-1.18)	0.000
Sung K C,2015 [22]	Both(60%)	1.07(0.95-1.20)	0.266
Li Y,2016 [23]	Male	1.13(1.10-1.18)	0.000
Li Y,2016 [23]	Female	1.19(1.04-1.35)	0.009
Hozawa A,2006 [27]	Male	0.97(0.87-1.08)	0.543
Hozawa A,2006 [27]	Female	1.17(1.03-1.33)	0.015
Total	Total	1.10(1.08-1.11)	0.000

Notes:In the study-specific dose-response analysis, HRs were significant in 9 studies ($P < 0.05$) and not significant in 2 studies ($P > 0.05$). The total HR was significant with substantial heterogeneity (I2 = 49.3% , P= 0.032).
