# Supplementary material for: Gamma-glutamyltransferase and risk of cardiovascular mortality: A dose-response meta-analysis of prospective cohort studies
Source: PLoS One. 2017 Feb 23;12(2):e0172631. doi: 10.1371/journal.pone.0172631 (PMC5322906; doi:10.1371/journal.pone.0172631)
Supplement: S2 Table — (PDF) [file pone.0172631.s004.pdf]

S2 Table. Dose-response meta-analysis on hazard ratios (HRs) for CV mortality per 10U/L increase in GGT, each study removed in turn.

Study removed		Pooled HR (95% CI) of remaining studies	P value	Test for heterogeneity	
First author, year	Gender (men %)			I^2 (%)	P value
Ruttmann E-M,2005 [17]	Male	1.11 (1.10, 1.13)	0	29.90%	0.17
Ruttmann E-F,2005 [17]	Female	1.10 (1.08, 1.11)	0	63.00%	0.004
Wannamethee S G,2008 [18]	Male	1.09 (1.08, 1.11)	0	62.80%	0.004
Breitling L P,2011 [19]	Male	1.09 (1.08, 1.11)	0	60.50%	0.007
Kengne A P-M,2012 [20]	Male	1.10 (1.08, 1.11)	0	62.90%	0.004
Kengne A P-F,2012 [20]	Female	1.09 (1.08, 1.10)	0	55.20%	0.017
Sung K C,2015 [22]	Both(60%)	1.10 (1.08, 1.11)	0	62.80%	0.004
Li Y-M,2016 [23]	Male	1.09 (1.08, 1.10)	0	55.20%	0.017
Li Y-F,2016 [23]	Female	1.09 (1.08, 1.11)	0	60.60%	0.007
Hozawa A-M,2006 [27]	Male	1.10 (1.08, 1.11)	0	53.20%	0.023
Hozawa A-F,2006 [27]	Female	1.09 (1.08, 1.11)	0	61.40%	0.006
